# Axin gene methylation status correlates with radiosensitivity of lung cancer cells

**DOI:** 10.1186/1471-2407-13-368

**Published:** 2013-08-02

**Authors:** Lian-He Yang, Yang Han, Guang Li, Hong-Tao Xu, Gui-Yang Jiang, Yuan Miao, Xiu-Peng Zhang, Huan-Yu Zhao, Zheng-Fan Xu, Maggie Stoecker, Endi Wang, Ke Xu, En-Hua Wang

**Affiliations:** 1 Department of Pathology, First Affiliated Hospital and College of Basic Medical Sciences, China Medical University, Shenyang, Liaoning, China; 2 Department of Radiation Oncology, First Affiliated Hospital of China Medical University, Shenyang, Liaoning, China; 3 Department of Pathology, Duke University Medical Center, Durham, NC, USA; 4 Department of Radiology, First Affiliated Hospital of China Medical University, Shenyang, Liaoning, China

**Keywords:** Axin, Methylation, Proliferation, Invasiveness, Radiosensitivity

## Abstract

**Background:**

We previously reported that Axin1 (Axin) is down-regulated in many cases of lung cancer, and X-ray irradiation increased Axin expression and inhibited lung cancer cells. The mechanisms, however, were not clear.

**Methods:**

Four lung cancer cell lines were used to detect the methylation status of Axin with or without X-ray treatment. Real-time PCR was used to quantify the expression of Axin, and western blot analysis was applied to measure protein levels of Axin, β-catenin, Cyclin D1, MMP-7, DNMTS, MeCP2 and acetylated histones. Flow cytometric analysis, colony formation assay, transwell assay and xenograft growth experiment were used to study the biological behavior of the cells with hypermethylated or unmethylated Axin gene after X-ray treatment.

**Results:**

Hypermethylated Axin gene was detected in 2 of 4 cell lines, and it correlated inversely with Axin expression. X-ray treatment significantly up-regulated Axin expression in H446 and H157 cells, which possess intrinsic hypermethylation of the Axin gene (*P*<0.01), but did not show up-regulation in LTE and H460 cells, which have unmethylated Axin gene. 2Gy X-ray significantly reduced colony formation (from 71% to 10.5%) in H157 cells, while the reduction was lower in LTE cells (from 71% to 20%). After X-ray irradiation, xenograft growth was significantly decreased in H157 cells (from 1.15 g to 0.28 g) in comparison with LTE cells (from 1.06 g to 0.65 g). Significantly decreased cell invasiveness and increased apoptosis were also observed in H157 cells treated with X-ray irradiation (*P*<0.01). Down-regulation of DNMTs and MeCP2 and up-regulation of acetylated histones could be detected in lung cancer cells.

**Conclusions:**

X-ray-induced inhibition of lung cancer cells may be mediated by enhanced expression of Axin via genomic DNA demethylation and histone acetylation. Lung cancer cells with a different methylation status of the Axin gene showed different radiosensitivity, suggesting that the methylation status of the Axin gene may be one important factor to predict radiosensitivity of the tumor.

## Background

Axin is an important factor in c-Jun N-terminal kinase (JNK), p53, Wnt and other signal transduction pathways [1,2], and decreased expression of Axin has been noted in many malignant tumors, including gastric, colorectal, breast, and other cancers [1,3,4]. We have demonstrated that Axin is down-regulated in many cases of lung cancer, and a low level of Axin expression correlates directly with disease progression and poor prognosis in patients with lung cancer [5]. The mechanism of down-regulation of Axin in cancer patients is not entirely clear at the present time. Although mutations in the Axin gene have been detected and implicated in a few types of malignant tumors, the mutation rate is low and sporadic, and the hot spots of the mutations have not been identified in any specific type of malignant tumor [6-11]. These sporadic mutations hardly explain the universal decrease in the expression of Axin in many cases of cancer [12]. It is well known that hypermethylation of certain tumor suppressor genes could result in down-regulation or even silencing of these genes, leading to the development and progression of malignant tumors [13]. By analyzing genomic sequences we noted that the Axin gene is rich in CpG islands promoter region and in some introns, and thus, hypothesize that the decreased expression of Axin in lung cancer cases may be caused by hypermethylation.

In a previous study, we reported that X-ray irradiation significantly up-regulates Axin expression in some fresh non-small cell lung cancer (NSCLC) tissues (5/10) [14], but the underlying molecular mechanism for this regulation is unknown. Interestingly, X-ray irradiation has been shown to induce demethylation of the whole genome by inhibiting DNA methyltransferases (DNMTs) and methyl-binding protein 2 (MeCP2) [14-21]. These previous studies raise the possibility that X-ray irradiation triggers apoptosis of lung cancer cells via demethylation- and acetylation-mediated up-regulation of the Axin gene by inhibiting DNMTs and MeCP2 [22].

In order to confirm our hypothesis, we assessed the methylation status of the Axin gene and investigated transcriptional expression of Axin. In addition, we studied the effects of X-ray irradiation on expression of Axin, DNMTs, and MeCP2, its effect on the methylation status of the Axin gene, and the associated changes in cell proliferation, invasiveness, apoptosis and tumor progression.

## Methods

### Cell culture and X-ray treatment

Three cell lines of Non-small cell lung cancer (NSCLC), including LTEP-a-2 (LTE, adenocarcinoma), NCI-H157 (H157, adenocarcinoma) and NCI-H460 (H460, large cell carcinoma) and one cell line of small cell lung carcinoma (SCLC) NCI-H446 (H446) were cultured in plastic flasks with RPMI 1640 medium (GBICO Inc., NY, USA) containing 10% fetal calf serum (GBICO Inc., NY, USA) at 37°C in a humidified atmosphere (5% CO2 and 95% air). The plastic flasks with lung cancer cells were treated with X-ray irradiation using a linear accelerator (Primus, Siemens, Germany) with a dose of 1Gy and 2 Gy, respectively, according to the previous study [14]. X-ray irradiation was delivered soon after the cell density reached 70-80%. Untreated lung cancer cells were used as a control. After irradiation, the cells were harvested at the appropriate time points and reserved in a refrigerator (−80°C) before being processed for further analysis. As previously demonstrated, lung cancer cell lines with different histological types usually show different radiosensitivity. In order to exclude an influence from histological type, two adenocarcinoma cell lines with different methylation statuses and expression levels (H157 and LTE) were used in in vitro and in vivo experiments to study the effect of X-ray irradiation.

### Nested MSP, Real-time RT-PCR and western blot analysis

The genomic DNA from lung cancer cells treated with or without X-ray irradiation were isolated by using a DNA extraction kit (TIANGEN BIOTECH BEIJING CO., LTD) according to the manufacturer’s instructions. Aliquots of DNA samples were treated with a DNA methylation kit (ZYMO RESEARCH Inc., USA). Hypermethylated Axin gene was defined when a distinctive amplicon was demonstrated on gel electrophoresis after methylation specific PCR (MSP), while unmethylated Axin gene was designated when no distinctive amplicon was seen after methylation specific PCR and clear amplicon was produced by unmethylation specific PCR. The primers for PCR reactions are listed in Table 1.

**Table 1:** Sequence and reaction conditions of nested MSP and real-time PCR primer

**Name**	**Sequence**	**Length**	**TM**	**Cycle**
***MSP primers***				
Axin promoter (Outside primer)	F:5′GGAGGTTTTGGTTTTTTAGAGAGYGGAG 3′	298 bp	55°C	35
	R: 5′AAACCCTAACCATCCCTACCTACCRACC 3′			
Axin promoter methylation	F: 5′GTAGGTTTTTGGAATGGTCGC 3′	144 bp	55.7°C	35
	R: 5′ACTAAACAAAAAACCCCGAA 3′			
Axin promoter unmethylation	F: 5′ GTAGGTTTTTGGAATGGTTGTGG 3′	144 bp	55.2°C	35
	R: 5′ ACTAAACAAAAAACCCCAAA 3′			
Axin intron 1 (Outside primer)	F: 5′TGTTTATAATTTTAGTTATTTGGGAAGGT 3′	283 bp	55°C	35
	R: 5′ACCCCTTATTTTTACTCACACTTCTATT 3′			
Axin intron 1 methylation	F:5′ GTTGAGGTAGGAGAATCG 3′	222 bp	59.3°C	35
	R:5′ TCTTCAGGAAAAATCTCG 3′			
Axin intron 1 unmethylation	F:5′ GTTGAGGTAGGAGAATAG 3′	222 bp	59°C	35
	R:5′ TCTTCAGGAAAAATCTAG 3′			
Axin intron 2 (Outside primer)	F: 5′ GGATAAATATAGAAAAGGGTTAGGAATG 3′	362 bp	57.5	35
	R: 5′ ATAAACTAAAAAACTCCTCAAATACCAC 3′			
Axin intron 2 methylation	F:5′ AAGTGAGAGTTTAGGTAGAGGAGGC3′	238 bp	58°C	35
	R:5′ CAAAAAAACTAAATACCTATAACCG 3′			
Axin intron 2 unmethylation	F:5′ GAGAGTTTAGGTAGAGGAGGT 3′	234 bp	59.5°C	35
	R:5′ CAAAAAAACTAAATACCTATAACCA 3′			
***Real-time PCR primers***				
Axin primer	F: 5′- TCACCCTGGGCCAGTTCAA -3′			
	R: 5′-CAGTCAAACTCGTCGCTCACTTTC -3′			
β-actin primer	F:5′- AGCACAGAGCCTCGCCTTTG -3′			
	R:5′- ACATGCCGGAGCCGTTGT -3′			

Total RNA was isolated from lung cancer tissues and cultured cells with TRIzol Reagent (Invitrogen). Real-time RT-PCR (Fluorescent dye method, ABI, 7900HT) was performed to evaluate the transcripts of Axin. The experiments were performed according to the manufacturer’s instructions (SYBR® Premix Ex Taq™, Takara Bio). Each assay was repeated three times. The PCR primers are listed in Table 1.

Mouse monoclonal antibody against DNMT1 (H-12, 1:300, Santa Cruz Biotechnology, Santa Cruz, CA, USA.), β-actin (sc-8432, 1:500, Santa), β-catenin (562505, 1:800, BD Transduction Laboratories, NJ, USA), and acetylated histone H3 (H3-ab47915, 1:500, Abcam, Cambridge, MA, UK) and rabbit polyclonal antibody against acetylated histone H4 (H4-06-598, 1:500, Upstate Biotechnology Incorporated, NY, USA), DNMT3B (ab2851, 1:500, Abcam), Axin (06–922, 1:500, Upstate), MeCP2 (ab2828, 1:500, Abcam), Cyclin D1 (H-295, 1:500, Santa) and MMP-7 (sc-30071, 1:500, Santa) were used in Western blot analysis. The protein bands on the membrane were visualized using ECL (Pierce, Rockford, IL, USA) and quantified using the DNR Bio-Imaging System. The relative protein levels were calculated by normalizing to the amount of β-actin. The experiment was repeated three times, and a mean value was presented.

### Colony formation, matrigel invasion and flow cytometric analysis

Colony Formation: 500 cells were grown in a 60 mm dish with culture medium. The cells were treated with X-ray irradiation at doses of 1 Gy or 2 Gy, respectively, after 12 hours of incubation. The cells were then continuously cultured until visible colonies were formed (14 days). Only those containing ≥50 cells were counted. The rate of colony formation was indicated by the ratio of the number of clones over the number of seeded cells. The experiment was repeated three times, and a mean value was presented.

Matrigel cell invasion assay: Briefly, in each upper chamber, 5×10^5^ cells (with or without X-ray irradiation) were grown in serum-free culture medium. The lower chambers were filled with RPMI 1640 medium containing 10% fetal calf serum. After being incubated for 24 hours, the cells that migrated through the pores were fixed with methanol for 30 minutes and stained with hematoxylin. For each filter, the number of cells was counted microscopically in 5 random fields under a 200×magnification. The experiment was repeated three times, and a mean value was presented.

Flow cytometric analysis for cell apoptosis: Cells were collected at 72 hours after X-ray treatment and then immediately stained with the Annexin V-FITC/PI double staining kit (Keygene Biotechnology) before being analyzed by the FACSCalibur Flow Cytometer with Cell Quest 3.0 software (BD) to determine the level of cell apoptosis. The experiment was repeated three times, and a mean value was presented.

### Xenograft to nude mice

Four-week-old male BALB/c nude mice were obtained from the animal facility. All the mice were handled in strict accordance with the recommendations in the Guide for the Care and Use of Laboratory Animals of the National Institutes of Health [23]. The protocol was approved by the Committee on the Ethics of Animal Experiments of the China Medical University. All efforts were made to minimize suffering of the experimental animals. The mice were randomly divided into 4 groups (5 mice in each group, weight 15.2-16.8 grams). Each mouse was inoculated subcutaneously in the right axilla with 5×10^6^ human lung cancer cells suspended in 0.2 ml sterile PBS. The large dimension (L) and short dimension (W) of the subcutaneous nodules were measured with a vernier caliper every 3 days, and the tumor volume was calculated by the formula, V = W^2^ × L × π/6, before being plotted into the growth curve for each group. Four weeks after inoculation, the mice were sacrificed, and the tumor nodules from each mouse were completely excised and measured. The rate of tumor growth inhibition (%) was calculated according to the formula: (mean tumor weight of control group-mean tumor weight of X-ray irradiation group)/mean tumor weight of control group×100%.

### Statistical analysis

SPSS version 13.0 for Windows was utilized to analyze the data. The Mann–Whitney *U* test and Student’s *t* test were used to examine the statistical difference of experimental data between the groups.

## Results

### Effect of X-ray irradiation on axin mrna expression and methylation in lung cancer cells with hypermethylated or unmethylated Axin gene

**Figure 1:**
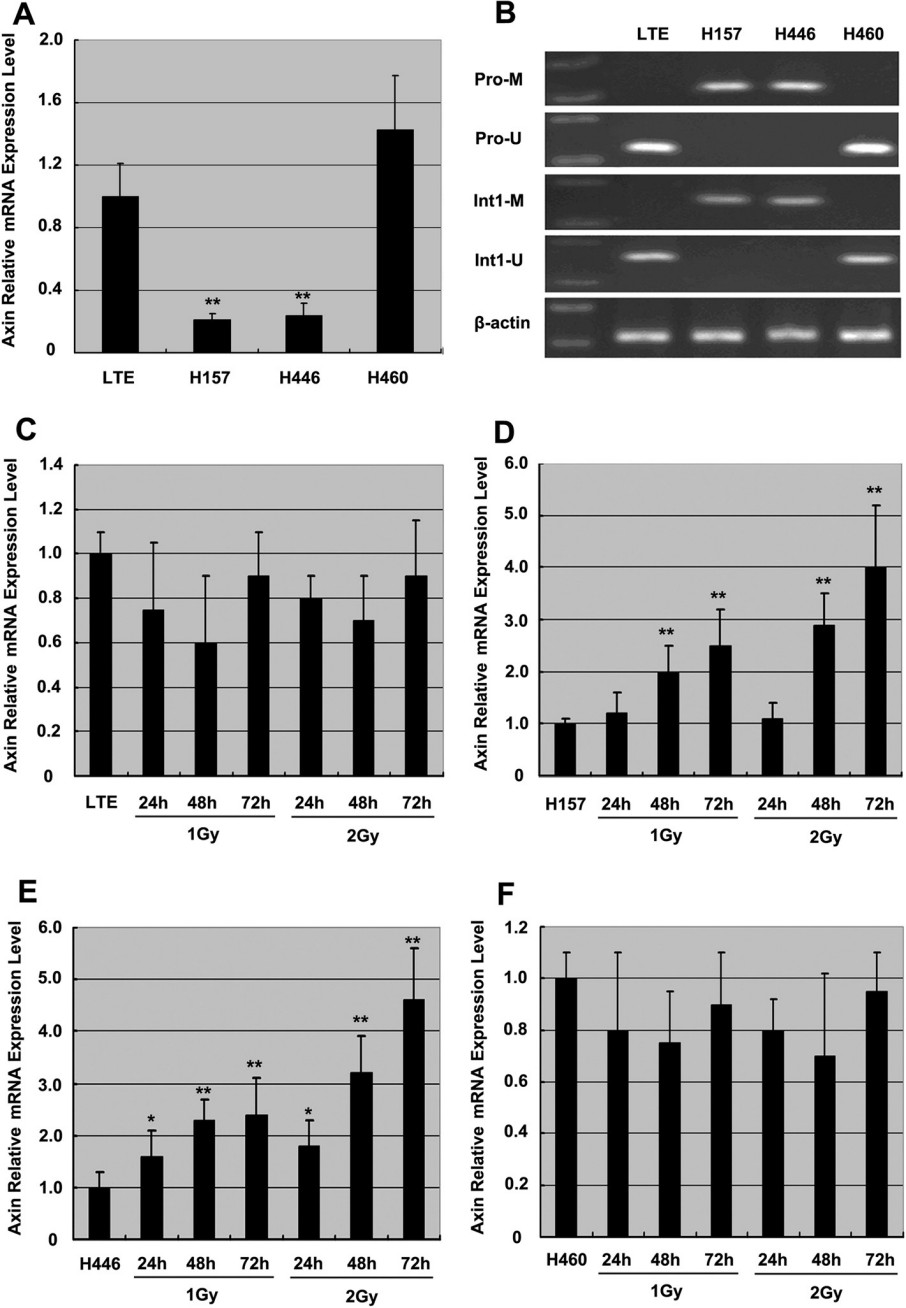
**X-ray induced over-expression of the Axin gene in lung cancer cell lines with hypermethylated Axin gene. A** and **B** show the methylation status of the promoter and first intron of the Axin gene and levels of Axin transcripts in 4 lung cancer cell lines. Note low Axin mRNA expression **(A)** in H157 and H446 cells, which exhibit a hypermethylated Axin gene** (B)**. Real-time PCR shows no up-regulation of Axin mRNA in LTE cells and H460 cells **(C** and **F)** but a marked increase in the Axin transcripts in H157 **(D)** and H446 **(E)** cell lines after X-ray treatment. Note time and dose dependent increase in Axin transcripts after the treatment in both H157 **(D)** and H446 **(E)** cell lines. Pro-M, amplification for the methylated Axin gene promoter; Pro-U, amplification for the unmethylated Axin gene promoter; Int1-M, amplification for the methylated first intron of the Axin gene; Int1-U, amplification for the unmethylated first intron of the Axin gene. * *P*<0.05, and ** *P*<0.01, in comparison with the group without X-ray irradiation.

Nested MSP showed that the promoter and first intron regions of the Axin gene are hypermethylated in H157 and H446 cells but unmethylated in LTE and H460 cells, and correspondingly, Real-time RT-PCR demonstrated that H157 and H446 cells had a mean level of Axin mRNA significantly lower than LTE and H460 cells (Figure 1A and B) (*P*<0.01). This result suggests that hypermethylated Axin gene correlated inversely with Axin expression. Then all cell lines were treated with X-ray irradiation. Axin mRNA was apparently up-regulated in H157 and H446 cells that have hypermethylated Axin gene but not in LTE and H460 cells that have unmethylated Axin gene (Figure 1C-F). Interestingly, X-ray irradiation in H157 (Figure 1D) and H446 cells (Figure 1E) seems to demonstrate time dependent and dose dependent increases of Axin transcripts, with a more significant increase noted at the 72 hour point (P<0.01) and with 2 Gy. This time and dose dependent fashion of up-regulation of the Axin gene was not observed in LTE and H460 cells. Axin mRNA was not increased after X-ray irradiation in LTE (Figure 1C) or H460 cells (Figure 1F). These results suggest that X-ray irradiation could possibly up-regulate Axin expression in the cells with hypermethylated Axin gene but not in the cells with unmethylated Axin gene.

**Figure 2:**
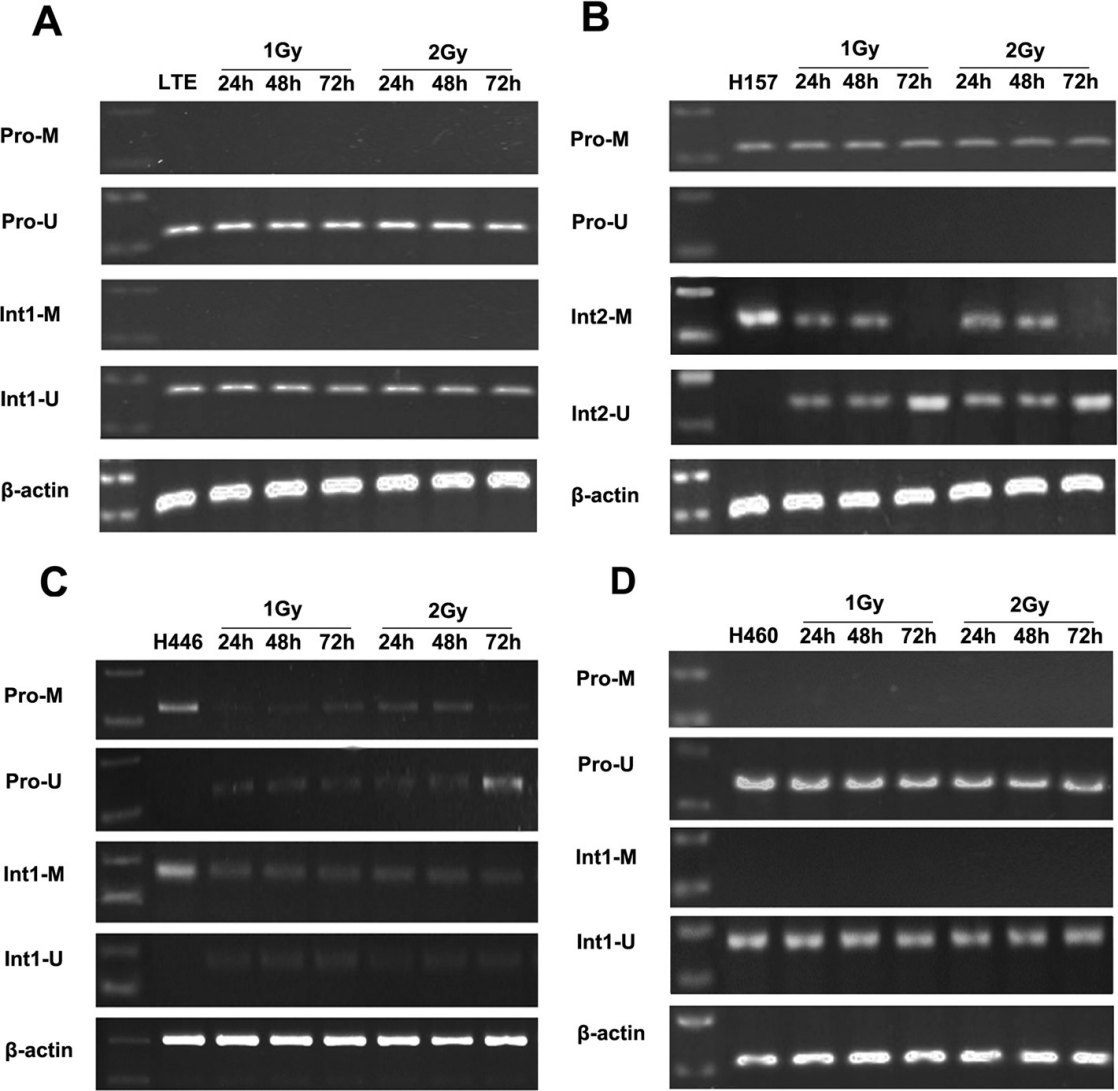
**The effect of X-ray irradiation on methylation status of the Axin gene in lung cancer cell lines.** There is no obvious change in unmethylated status of the Axin promoter and first intron in the LTE **(A)** and H460 **(D)** cell lines after X-ray irradiation, while significant demethylation could be detected in the second intron of H157 cells** (B)** and the promoter and first intron in H446 cells **(C)**.

MSP demonstrated that there was no change of the unmethylated status of LTE and H460 cells after X-ray irradiation (Figure 2A and D), while in contrast, methylation of the Axin gene was decreased along with an associated increase in unmethylated sequences in the promoter and first intron regions of the H446 cell line, which has an intrinsic hypermethylated Axin gene (*P*<0.05) (Figure 2C). Although demethylation of the promoter and first intron regions in the H157 cell line was not detected (data not shown), a significant demethylation in the second intron region could be observed in this cell line after X-ray irradiation (*P*<0.05) (Figure 2B). These results suggest that X-ray irradiation may induce Axin expression via demethylating the DNA in lung cancer cells.

### X-ray-induced DNMTs down-regulation and acetylated histone up-regulation correlated with Axin gene methylation status and expression

**Figure 3:**
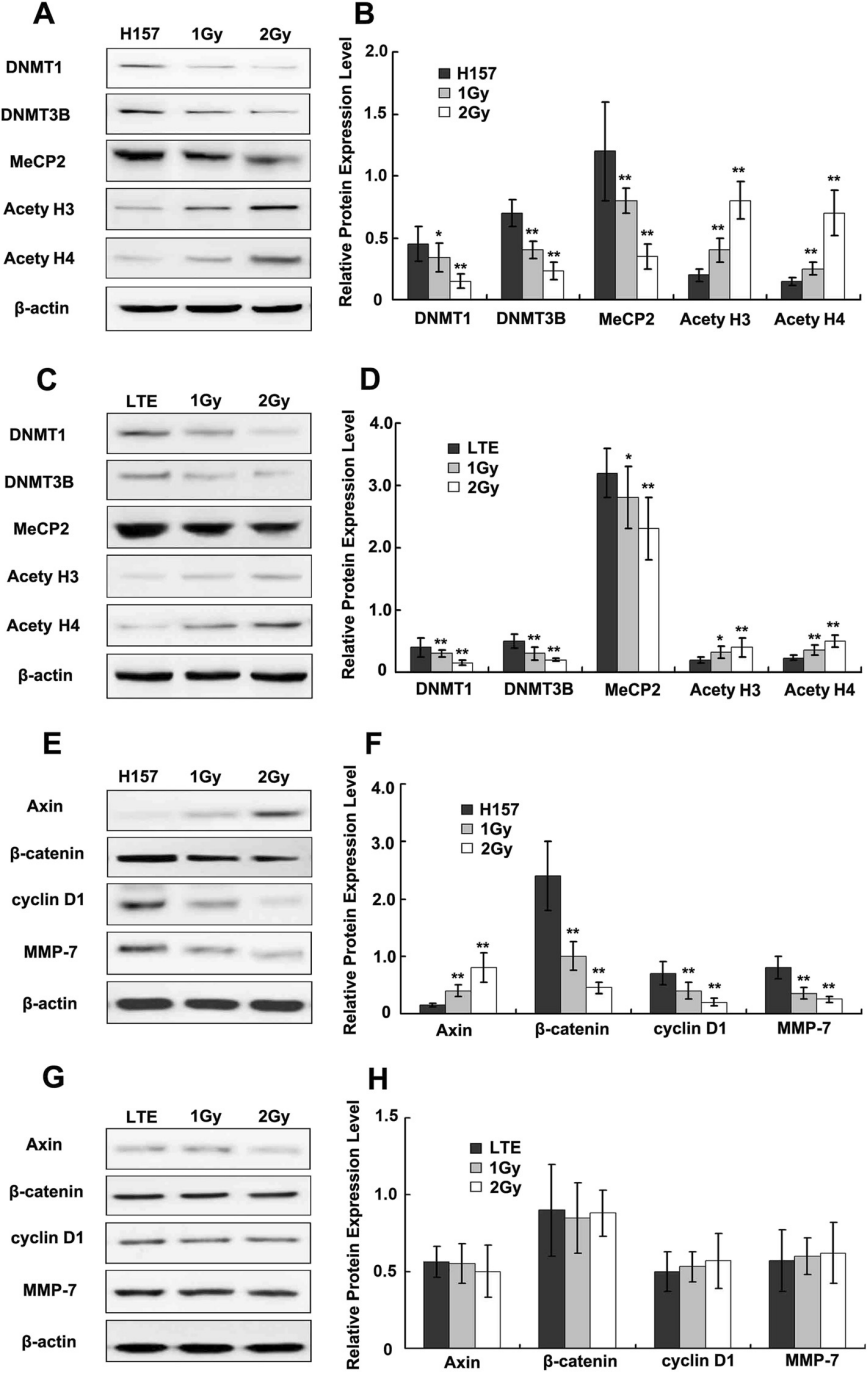
**The effect of X-ray irradiation on DNMTs, MeCP2, acetylated histones and factors of the Wnt signaling pathway.** Western blot analysis shows the effects of X-ray irradiation on the expression of DNMTs, MeCP2, acetylated histones, Axin, β-catenin, Cyclin D1 and MMP-7 at 24h after X-ray treatment. DNMT1, DNMT3B and MeCP2 are significantly down-regulated in H157 cells, and acetylated histone H3 and H4 are up-regulated in H157 cells **(A**, **B)** is the histogram of A. DNMT 1 and 3B are also down-regulated significantly in LTE cells. Slightly decreased MeCP2 expression and up-regulation of acetylated H3, H4 are noted in LTE cells **(C** and **D)**, with the degree of change less significant than in H157 cells. Increased Axin expression and decreased β-catenin, Cyclin D1 and MMP-7 expression are noted in the H157 cell line after X-ray irradiation **(E** and **F)**, but no significant change in these factors are detected in LTE cells **(G** and **H)**. Note a dose dependent pattern of the changes in both cell lines, with more prominent changes in the H157 than in the LTE cell line. * *P*<0.05, ** *P*<0.01.

It has been reported that X-ray irradiation could induce demethylation by inhibiting DNMTs and MeCP2 [14-21]. DNA methylation is regulated by DNMTs, a family of enzymes catalyzing transfer of methyl groups to genomic DNA [13]. We examined the protein levels of DNMT1 and 3B at 24 hours after 1 Gy and 2 Gy X-ray irradiation, respectively, in two NSCLC cell lines: H157 (hypermethylated Axin gene) and LTE (unmethylated Axin gene). Both DNMT1 and DNMT3B were significantly down-regulated in the two cell lines (Figure 3A-D) (*P*<0.01), with more significant effects seen in the H157 cell line than in the other.

MeCP2 could bind to DNA methyl groups and recruit histone deacetylase (HDAC), resulting in histone deacetylation, chromatin condensation, and consequently, transcriptional inactivation of the genes [13]. Therefore, we examined the expression of MeCP2 and acetylated histones in H157 cells and demonstrated a decrease in MeCP2 protein associated with a marked increase in acetylated histone H3 and H4 (Figure 3A and B, *P*<0.01). Decreased MeCP2 protein and increased acetylated H3 and H4 proteins could also be detected in LTE cells (Figure 3C and D, *P*<0.05), but the effects were less significant than those observed in H157 cells. Interestingly, the decreases in DNMT1, DNMT3B and MeCP2 proteins were present in a dose dependent fashion after treatment with X-ray irradiation. The increases in acetylated H3 and H4 in both cell lines, with more significant effects seen in the H157 cell line, were also present in a dose dependent fashion after treatment with X-ray irradiation.

Given the insignificant demethylation of the Axin gene in the H157 cell line, the X-ray induced increase in Axin transcripts in this cell line with intrinsic hypermethylated Axin gene may be partially explained by inhibition of MeCP2, which could cause decreased histone deacetylase, and thus, lead to transcriptional activation of the Axin gene via histone acetylation.

Significant up-regulation of the Axin protein could be detected in H157 cells but not in LTE cells after 1 Gy or 2 Gy X-ray irradiation (Figure 3E-H). β-catenin is a key positive regulator of the Wnt pathway, while Cyclin D1 and matrix metalloproteinase 7 (MMP-7) are important downstream factors of the Wnt signal pathway, which correlates with cell proliferation and invasion [22]. In this study, all three factors were significantly down-regulated in the H157 cells at 24 h (Figure 3E and F, *P*<0.01) but none were in LTE cells after X-ray irradiation (Figure 3G and H). These results suggest that X-ray irradiation could inhibit the Wnt signal transduction pathway probably via enhanced expression of the Axin gene. It is well known that activation of the Wnt signal transduction pathway significantly correlates with proliferation and invasion of tumor cells; therefore, we evaluated the change of the biological behavior in lung cancer cells with hypermethylated or unmethylated Axin gene.

### X-ray irradiation significantly inhibited growth and invasiveness of the lung cancer cells with hypermethylated Axin gene in in vitro and in vivo experiments

To investigate the effect of X-ray irradiation mediated Axin up-regulation on lung cancer cells and exclude the influence of different histological types of lung cancers, two cell lines with the same histological type (adenocarcinoma), H157 and LTE, were used to perform in vitro and in vivo experiments.

**Figure 4:**
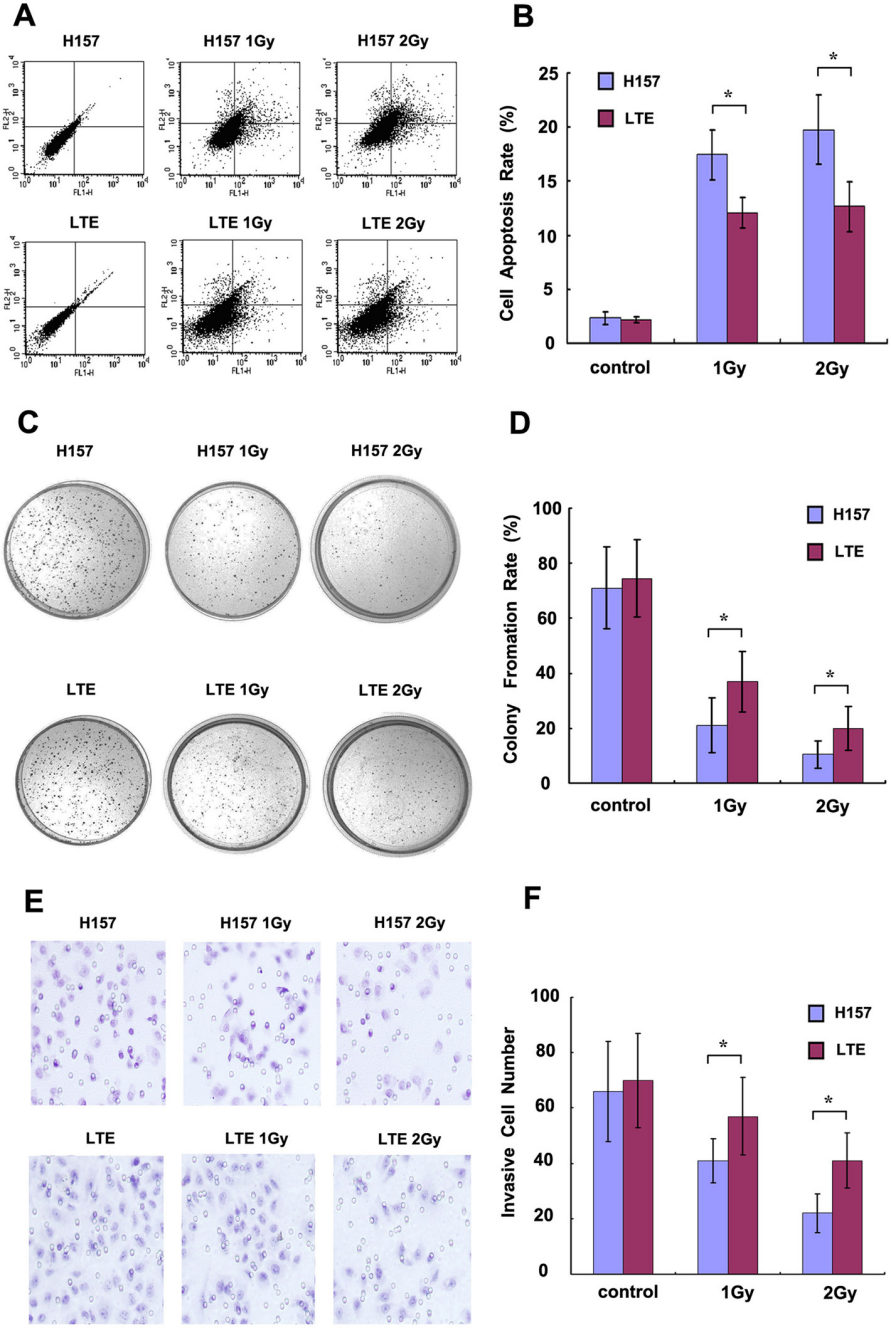
**The effect of X-ray irradiation on lung cancer cells with hypermethylated or unmethylated Axin gene. A** shows the effect of X-ray irradiation on cell apoptosis in lung cancer cells by flow cytometric analysis. The histogram in **B** summarizes the statistical data from A. The rate of colony formation in H157 and LTE cells with or without X-ray irradiation are shown in **C**, and the histogram in **D** summarizes the statistical data from **C**. The invasive cell numbers in H157 and LTE cells (treated or untreated with X-ray) are presented in **E**, **F** is the histogram of **E**. The cell apoptosis rate, colony formation rate and cell invasiveness are markedly changed when H157 and LTE cell lines are treated with X-ray irradiation, with a more prominent inhibitory effect seen in the H157 cell line than in the LTE cell line **(A-F)**. * *P*<0.05, comparison between the two cell lines.

We previously reported that X-ray mediated Axin up-regulation could induce apoptosis in lung cancer [14]. In this study, flow cytometric analysis for cell apoptosis (Figure 4A and B) demonstrated that the apoptosis rate in H157 cells was markedly increased after X-ray irradiation, and the effect of the irradiation was significantly stronger than that in the LTE cell line (1 Gy group: 17.45%±2.32% versus 12.06%±1.44%, and 2 Gy group: 19.79%±2.11% versus 12.66%±1.71%; *P*<0.05). The efficacy of colony formation in the H157 cells was 71% for the control group, 21% for 1 Gy irradiation and 10.5% for 2 Gy irradiation (Figure 4C and D). In contrast, X-ray treatment seemed to show less effect in the LTE cell line, with the efficacy of colony formation being 74.5%, 37% and 20% for the control, 1 Gy and 2 Gy irradiation, respectively (Figure 4C and D, *P*<0.05). Similarly, transwell cell invasive experiments (Figure 4E and F) showed that the invasive cell number of the H157 cell line was significantly decreased after irradiation, and as noted in the colony formation assay, the extent of X-ray effect was much more significant in H157 cells than in LTE cells in both dose groups (1 Gy group: 41±9 versus 57±12, 2 Gy group: 22±6 versus 41±10; *P*<0.05). There is no significant difference of cell apoptosis, cell invasiveness and colony formation between the two cell lines without irradiation.

This data provides evidence that X-ray irradiation significantly inhibits malignant behavior in lung cancer cells that have intrinsic hypermethylation of the Axin gene, but its effect in cancer cells with unmethylation of the gene seems to be less prominent. Therefore, we hypothesize that the lung cancer cells with hypermethylation of the Axin gene may be more sensitive to X-ray irradiation, and the cancer cells exposed to irradiation may have a disadvantage of xenograft growth in vivo over cell lines with unmethylation of this gene.

**Figure 5:**
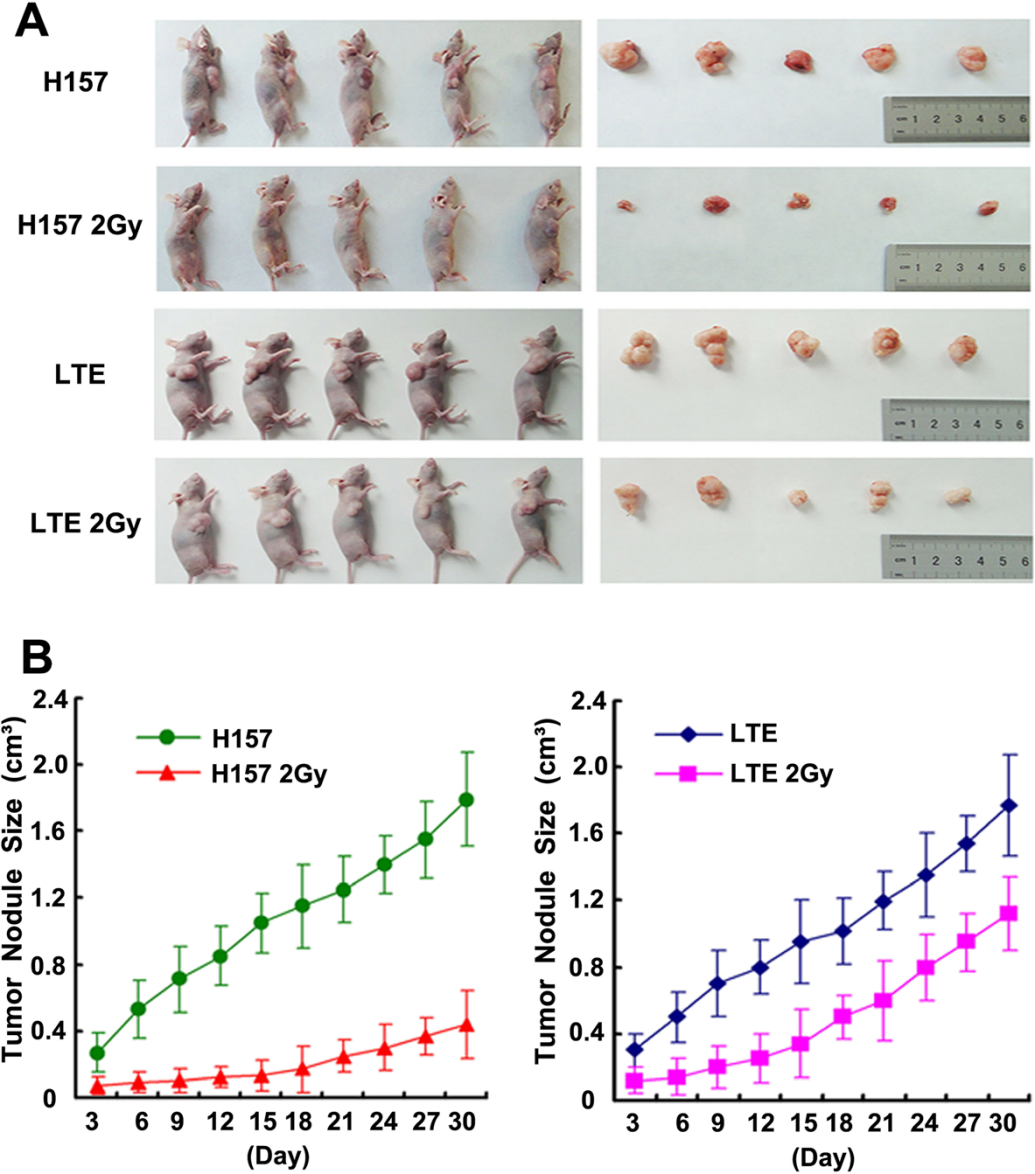
**The comparison of xenograft tumor growth between the cell line with a hypermethylated Axin gene (H157) and the cell line with an unmethylated Axin gene (LTE) after X-ray treatment.** Figure **A** shows asignificant decrease in tumor mass in the mice transplanted with X-ray treated cancer cells, particularly in the mice with treated H157 cells in comparison with each control. Figure **B** shows the curves of time dependent tumor growth. Note X-ray treatment significantly suppresses the growth of tumor xenografts, with a more significant effect noted in H157 cells than in LTE cells.

H157 and LTE cells with or without X-ray irradiation (2 Gy) were inoculated into nude mice, respectively, and the tumors were completely excised 4 weeks later (Figure 5A). The weight of tumor was markedly reduced in H157 cells receiving irradiation from 1.15 ± 0.37g to 0.28 ± 0.08 g (*P*<0.01), and the size of tumor was decreased from 1.77 ± 0.63 cm^3^ to 0.44 ± 0.12 cm^3^ (*P*<0.01). The rate of tumor inhibition in the H157 cell line (76.63%, weight) was significantly higher than in the LTE cell line (38.5%, from 1.06 ± 0.22 g to 0.65 ± 0.21 g, *P*<0.01). There is no statistically significant difference in the rates of xenograft growth between the 2 cell lines without irradiation in tumor size and growth, but the difference is statistically significant between H157 cells with irradiation and LTE cells with irradiation in tumor size and growth (*P*<0.01) (Figure 5B). While X-ray irradiation showed the suppression of tumor growth in both cell lines, the extent of suppression in H157 cells was much more prominent than in LTE cells.

### Combined use of 5-Aza and TSA significantly up-regulate Axin transcripts in cells with hypermethylated Axin gene

**Figure 6:**
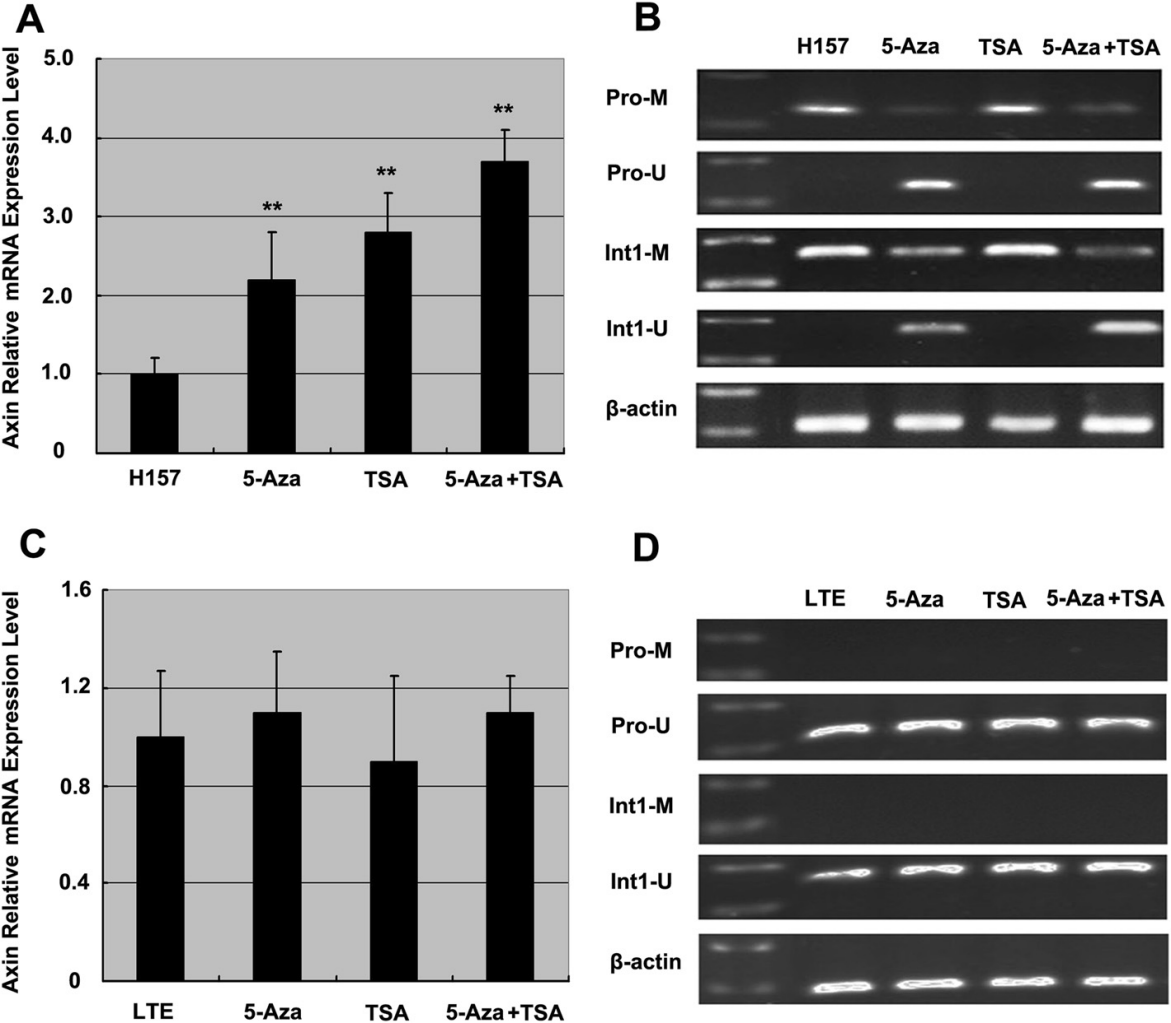
**The effect of 5-Aza and TSA treatment on lung cancer cell lines with hypermethylated Axin gene or unmethylated Axin gene.** The Axin mRNA expression is significantly up-regulated in H157 cells after 5-Aza treatment or TSA treatment. Further increases in Axin transcripts are detected after combined use of 5-Aza and TSA in the H157 cells **(A** and** B)** but not in LTE cells **(C** and **D)**. ** *P*<0.01, in comparison with control group (cells with no treatment).

Demethylation agent 5-Aza-2-Deoxycytidine (5-Aza, Sigma, 10 μM, 72 h) and deacetylase inhibitor TSA were used, and transcripts of the Axin gene were measured. Significant demethylation and increased Axin transcripts could be detected in H157 cells after 5-Aza treatment (*P*<0.01) (Figure 6A and B). When Trichostatin A (TSA, Sigma, 300 nmol/L, 24 h), an inhibitor of histone deacetylase, was used, the Axin mRNA expression was also up-regulated significantly (*P*<0.01) with no altered level of Axin gene methylation (Figure 6A and B). An additional increase in Axin transcripts was noted with combined use of 5-Aza and TSA in H157 (Figure 6A and B), suggesting a synergistic effect of demethylation and acetylation. In contrast, neither 5-Aza treatment nor TSA treatment could significantly up-regulate Axin expression in LTE cells and neither showed effects on methylation status of the Axin gene (Figure 6C and D).

## Discussion

It has been reported that X-ray irradiation significantly reduces the number of 5-methylcytosines in genomic DNA of cultured cell lines [15,17-21,24-27]. To our knowledge, little is known about the epigenetic changes and alterations in expression of a specific gene after X-ray irradiation. In the current study, we demonstrate that X-ray irradiation up-regulates Axin expression in lung cancer cell lines with hypermethylated Axin gene (H157). The increased cell apoptosis rate and decreased tumor growth in H157 cells (hypermethylated Axin gene) is more significant than in lung cancer cells with unmethylated Axin gene (LTE). Given the association of X-ray induced over-expression of the Axin gene with inhibition of xenograft tumor growth, the results in the current study suggest a linkage between X-ray induced up-regulation of the Axin gene and tumor cell apoptosis. 5-Aza and TSA treatment could up-regulate the expression of Axin in H157 cells but not in LTE cells. Based on our data and previous reports, we hypothesize that up-regulation of the Axin gene may be mediated by X-ray induced demethylation and acetylation of histone proteins adjacent to the gene by down-regulating DNMTs and MeCP2. However, due to the universal effects of X-ray irradiation on cells, the effects of irradiation on Axin gene expression and biological behavior in lung cancer cells may be influenced by other factors, and therefore, additional studies are needed to further elucidate the mechanisms.

We noted that no demethylation was detected in H157 cells at the promoter or in the first intron. Of note, the nested MSP used to test the methylation status in this study is sensitive, but it is not able to detect the methylation status of the Axin gene beyond the region covered by the primers applied. When we designed the primer for the second intron and performed the test, significant demethylation was detected in this cell line after X-ray irradiation, thus confirming our hypothesis. Unfortunately, the epigenetic changes of the entire Axin gene are currently unclear, and thus, the methylation statuses in the regions beyond the promoter and the first and second introns of the Axin gene, as well as their functional significance, are difficult to determine at the present time. In our future investigations, we plan to perform additional tests, including bisulfite sequencing of the entire noncoding sequence of the Axin gene in different lung cancer cell lines and to correlate the methylation status of the gene with the corresponding response to X-ray treatment in each cell line to confirm our hypothesis.

Our previous study demonstrated that over-expression of the Axin gene is associated with down-regulation of β-catenin and consequent inhibition of the Wnt signaling pathway, which is accompanied with inhibition of invasion and proliferation in lung cancer cells [28,29]. Therefore, we propose that the X-ray induced Axin up-regulation could be an indicator of increased radiosensitivity in certain lung cancers. In other words, methylation status of the Axin gene might serve as a pathologic marker in predicting radiosensitivity for lung cancer patients, with a possible increase in radiosensitivity in lung cancers with a hypermethylated Axin gene and a possible decreased in radiosensitivity in those with an unmethylated Axin gene. We also noted that LTE cells whose Axin was shown to be unmethylated exhibited a decrease in cell proliferation and invasion after X-ray irradiation compared to the control cells, suggesting that Axin demethylation is not the sole factor governing X-ray induced cell death. Nonetheless, our study demonstrates, via both in vitro and in vivo experiments, that the malignant biological behavior is suppressed by X-ray irradiation more significantly in the H157 cell line with hypermethylated Axin gene than in the LTE cell line with unmethylated Axin gene. We propose that different methylation statuses of Axin correlates with raidosensitivity of lung cancer cells, and the hypermethylated Axin gene may potentially serve as a molecular pathologic marker for radiotherapy in these patients. More lung cancer cell lines with hypermethylated or unmethylated Axin genes may be used in future assays to further test our hypothesis. The use of methylation status of the Axin gene as a therapeutic marker in the clinical setting remains to be verified by additional clinical analyses.

## Conclusions

The methylation status of the Axin gene inversely correlated with its expression in lung cancer cells with hypermethylation associated with a low expression of the gene. X-ray irradiation could up-regulate Axin in lung cancer cells with hypermethylated Axin gene, probably via DNMTs and MeCP2-acetylated histones. Lung cancer cells with different methylation status of the Axin gene showed different radiosensitivities, suggesting that hypermethylation of the Axin gene may be one of the important factors that predict radiosensitivity.

## Competing interests

The authors declare that they have no financial conflict of interests.

## Authors’ contributions

The work presented here was carried out in collaboration between all authors. EH W, ED W, GL and KX defined the research theme, designed methods and experiments. LH Y carried out the molecular genetic studies, participated in the sequence alignment and drafted the manuscript. YH and HT X carried out the western blot. YM, XP Z, HY Z, and ZF X carried out the In vivo and in vitro experiments, analyzed the data. MS and ED W proofread the manuscript. All authors read and approved the final manuscript.

## Pre-publication history

The pre-publication history for this paper can be accessed here:

http://www.biomedcentral.com/1471-2407/13/368/prepub
