# Correction to: Axin gene methylation status correlates with radiosensitivity of lung cancer cells

**DOI:** 10.1186/s12885-019-5491-x

**Published:** 2019-03-27

**Authors:** Lian-He Yang, Yang Han, Guang Li, Hong-Tao Xu, Gui-Yang Jiang, Yuan Miao, Xiu-Peng Zhang, Huan-Yu Zhao, Zheng-Fan Xu, Maggie Stoecker, Endi Wang, Ke Xu, En-Hua Wang

**Affiliations:** 1 Department of Pathology, First Affiliated Hospital and College of Basic Medical Sciences, China Medical University, Shenyang, Liaoning China; 2 Department of Radiation Oncology, First Affiliated Hospital of China Medical University, Shenyang, Liaoning China; 3 Department of Pathology, Duke University Medical Center, Durham, NC USA; 4 Department of Radiology, First Affiliated Hospital of China Medical University, Shenyang, Liaoning China

**Correction to: BMC Cancer 2013, 13:368**

**https://doi.org/10.1186/1471-2407-13-368**

Following publication of the original article [1], it has been brought to our attention that in Fig. 4a, the panel for LTE 2Gy was identical to the panel for LTE 1Gy. The correct panel for LTE 2Gy is now included in the new version of Fig. 4 shown below. We apologize for any inconvenience caused.

**Fig. 4:**
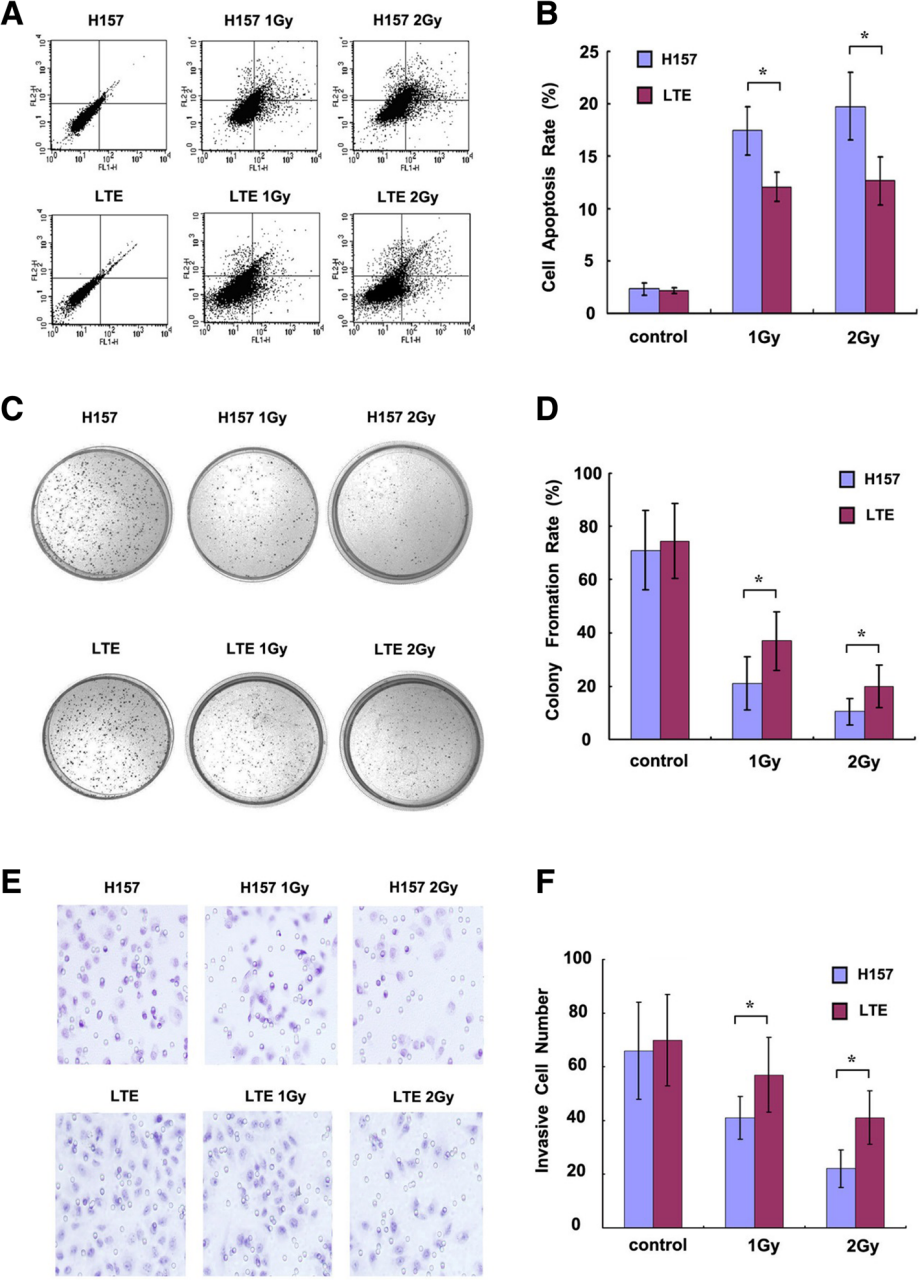
The effect of X-ray irradiation on lung cancer cells with hypermethylated or unmethylated Axin gene. **a** shows the effect of X-ray irradiation on cell apoptosis in lung cancer cells by flow cytometric analysis. The histogram in **b** summarizes the statistical data from **a**. The rate of colony formation in H157 and LTE cells with or without X-ray irradiation are shown in **c**, and the histogram in **d** summarizes the statistical data from **c**. The invasive cell numbers in H157 and LTE cells (treated or untreated with X-ray) are presented in **e, f** is the histogram of **e**. The cell apoptosis rate, colony formation rate and cell invasiveness are markedly changed when H157 and LTE cell lines are treated with X-ray irradiation, with a more prominent inhibitory effect seen in the H157 cell line than in the LTE cell line (**a- f**). * *P* < 0.05, comparison between the two cell lines
